# Effect of Expansive Admixtures on the Shrinkage and Mechanical Properties of High-Performance Fiber-Reinforced Cement Composites

**DOI:** 10.1155/2013/418734

**Published:** 2013-11-24

**Authors:** Won-Chang Choi, Hyun-Do Yun

**Affiliations:** ^1^Department of CAAE Engineering, North Carolina A&T State University, Greensboro, NC, 27411, USA; ^2^Department of Architectural Engineering, Chungnam National University, Daejeon 305–764, Republic of Korea

## Abstract

High-performance fiber-reinforced cement composites (HPFRCCs) are characterized by strain-hardening and multiple cracking during the inelastic deformation process, but they also develop high shrinkage strain. This study investigates the effects of replacing Portland cement with calcium sulfoaluminate-based expansive admixtures (CSA EXAs) to compensate for the shrinkage and associated mechanical behavior of HPFRCCs. Two types of CSA EXA (CSA-K and CSA-J), each with a different chemical composition, are used in this study. Various replacement ratios (0%, 8%, 10%, 12%, and 14% by weight of cement) of CSA EXA are considered for the design of HPFRCC mixtures reinforced with 1.5% polyethylene (PE) fibers by volume. Mechanical properties, such as shrinkage compensation, compressive strength, flexural strength, and direct tensile strength, of the HPFRCC mixtures are examined. Also, crack width and development are investigated to determine the effects of the EXAs on the performance of the HPFRCC mixtures, and a performance index is used to quantify the performance of mixture. The results indicate that replacements of 10% CSA-K (Type 1) and 8% CSA-J (Type 2) considerably enhance the mechanical properties and reduce shrinkage of HPFRCCs.

## 1. Introduction

Cement composites are widely used for the construction of civil infrastructure because of their high quality performance. However, cement composites that include short fibers typically are characterized by low tensile strength and quasibrittle behavior. A significant amount of research has been conducted to enhance the crack resistance and ductility of cement composites that contain short fibers [1–4]. Although the fracture toughness of cement composites can be improved by the inclusion of short fiber reinforcement, fiber-reinforced cement composites (FRCCs) exhibit tension-softening behavior after initial cracking. In the mid-1990s, Naaman and Reinhardt [5] proposed a new class of FRCC, that is, high-performance fiber-reinforced cement composite (HPFRCC). HPFRCC is a special class of FRCC that is able to resolve the problems associated with the post-crack, strain-softening behavior of tensile-loaded FRCCs. HPFRCCs are distinguished from ordinary FRCCs by their unique pseudo-strain-hardening and multiple cracking behaviors after initial cracks appear under uniaxial tension.

The ability of HPFRCC material to mitigate damage and dissipate energy greatly improves the mechanical performance of reinforced HPFRCC structures by preventing brittle failure and the loss of structural integrity, which are deficiencies often found in conventional reinforced concrete structures under excessive loading [6–10]. Experimental research has shown potential field applications that could benefit from the utilization of HPFRCC materials. Recently, HPFRCC materials were used for the Mihara Bridge (Hokkaido, Japan), the Grove Street Bridge (Ypsilanti, Michigan), and Pacific Tower Roppongi (Tokyo, Japan). It is expected that more structures will be designed using HPFRCC materials for critical structural elements in the near future [11].

However, to ensure the strain-hardening and multiple cracking behavior found in HPFRCCs, a low sand-to-binder (s/b) ratio and rich mixture without coarse aggregates are required in order to control the fracture toughness of the matrix [12]. Given these requirements, high shrinkage strain in HPFRCCs is probably their most disadvantageous property. Shrinkage generally leads to cracking, which typically compromises the structural integrity and durability of the structure [13]. The literature indicates that controlling the mixture proportions [14] and using shrinkage-reducing admixtures (SRAs) [15] and expansive admixtures (EXAs) [16] are effective methods to mitigate shrinkage in HPFRCCs.

Zhang et al. [14] investigated the effects of mixture parameters, such as water-to-binder (w/b) ratio, sand-to-binder (s/b) ratio, and cement types, such as Portland cement and composite cement that includes CaO, SiO_2_, and Al_2_O_3_ as their main components, on drying shrinkage as well as the tensile behavior of engineered cementitious composite (ECC), which is a kind of HPFRCC. Their test results indicate that the replacement of Portland cement by composite cement reduces the drying shrinkage of ECC with 1.7% polyethylene (PE) fibers.

Wang et al. [15] evaluated the effect of SRA on the surface tension, contact angle, and flexural behavior of both steel and polypropylene (PP) FRCCs. They found that the addition of 3% SRA by mass of water improves the flexural toughness of steel FRCC, whereas SRA used in PP FRCC is not effective in enhancing flexural toughness.

Cheung and Leung [16] investigated the effect of calcium sulfoaluminate (CSA) cement and SRA on the shrinkage of high-strength HPFRCC with w/b ratios of 0.19, 0.3, and 0.4. Their test results indicate that CSA is more effective in reducing shrinkage in HPFRCCs with higher w/b ratios, whereas SRA is more effective for HPFRCCs with lower w/b ratios. Furthermore, they found that hybrid CSAs and SRAs can significantly reduce the shrinkage of HPFRCC.

Şahmaran et al. [17] investigated the effect of using a replacement percentage of saturated lightweight fine aggregate (LWA) on the shrinkage and mechanical behavior of ECC. Their test results show that up to 20% replacement of normal weight silica sand with saturated LWA is very effective in reducing the autogenous shrinkage and drying shrinkage of ECC. They also reported that the autogenous shrinkage and drying shrinkage of ECC significantly decrease with an increase in fly ash content in the binder.

In this study, the effect of replacing cement with CSA EXA on the shrinkage and mechanical properties such as compressive, flexural, and direct tensile strength of 1.5% PE HPFRCC is investigated, and the proper replacement rate for the HPFRCC mixtures with respect to type of EXA is determined.

## 2. Experimental Program

### 2.1. Materials

FRCCs can mitigate the brittle nature of concrete by improving characteristics such as ductility, tensile capacity, and energy dissipation capacity. Li et al. [1, 18] report that the rich mix design of ECCs results in 160% more shrinkage than the shrinkage rate of 0.06% found for conventional concrete. Thus, research has been conducted to reduce this high shrinkage percentage by employing fiber, EXAs, and shrinkage-reducing agents. In related research, Lee and Yun [19] report that FRCC mixtures that contain 10% CSA as the EXA show improved performance in terms of compressive strength, flexural strength, and tensile strength due to the formation of ettringite, which is an expansive crystalline substance that forms when sulphate reacts with tri-calcium aluminates (C3A) and calcium hydroxide (Ca(OH)_2_). This admixture, that is, CSA EXA, occupies twice the volume of the original compounds.  Table 1 presents the major chemical components of two types of CSA EXA, that is, CSA-K (Type 1) and CSA-J (Type 2). As part of the chemical compositions of these two types of CSA EXA, CaO, SO_3_, and Al_2_O_3_ play a role in their expansion, high strength development, and early strength development, respectively. Large quantities of fine binder without coarse aggregate are used to control the fracture toughness of the cement matrix.

Some expansion admixtures contain F-CaO (or free CaO), which is more expandable than CaO bonded with other chemical compounds [20]. It is hypothesized that the mechanical properties of HPFRCC mixtures that contain EXA differ depending on whether the CSA EXA contains F-CaO or not. Research is needed to determine the proper replacement rate in the HPFRCC mixtures with respect to the type of EXA that is used. Thus, two types of EXA are examined in this study. One is a CSA EXA without any F-CaO and referred to as Type 1 (CSA-K). The other type is referred to as Type 2 (CSA-J) and is composed of 51% CaO and 16% F-CaO.

### 2.2. Mix Proportions and Mixing Procedure

In this study, the two types of EXA, Type 1 (CSA-K) and Type 2 (CSA-J), with a wide range of cement replacement percentages (0%, 8%, 10%, 12% and 14% by mass) are considered in the design of HPFRCC mixtures reinforced with 1.5% PE fiber by volume fraction. The design compressive strength of the HPFRCCs is 70 MPa. The material properties of the PE fiber are presented in Table 2.

Details regarding the HPFRCC mix designs are shown in Table 3. The individual specimens are identified in terms of amount of fiber, replacement rate of EXA, and type of EXA. For example, PE1.5-10-1 represents the specimen that is reinforced with 1.5% PE fiber and contains 10% Type 1 (CSA-K) EXA.

### 2.3. Specimen Preparation and Test Procedure

The mechanical properties, that is, shrinkage, compressive strength, flexural strength, and direct tensile strength, of the HPFRCC mixtures are examined in this study. In addition, crack width and development are examined to determine the effects of the EXAs on the HPFRCC mixtures. 

For the shrinkage tests, each prismatic specimen, 100 mm × 100 mm × 400 mm, was cured in an environmental chamber at 20 ± 1°C and relative humidity of 50 ± 1% after placement of the HPFRCC. Each specimen was demolded after 24 hours. The results for early age shrinkage within 24 hours and drying shrinkage after 24 hours were combined, and the internal shrinkage strain was measured by a shrinkage gauge embedded in the middle of each specimen.

For the compressive tests, three cylindrical specimens, 100 mm × 200 mm, for each type of HPFRCC mixture were tested in accordance with ASTM C39. Displacement gauges were installed on the sides and middle of each specimen.

For the flexural tests, three flexural beams were considered for each HPFRCC mixture. Four-point bending tests were conducted using a 200 kN universal testing machine (UTM) with displacement control of (0.5) mm/min.

For the direct tensile tests, five dumbbell-shaped specimens with 80 mm × 30 mm × 30 mm middle cross-sections were fabricated and tested in accordance with the recommendations for design and construction of HPFRCCs with multiple fine cracks by the Japan Society of Civil Engineers (JSCE) [21].

Finally, the formation of ettringite in the HPFRCC mixtures was examined using a scanning electron microscope (SEM) with 5 *μ*m resolution. The samples were prepared by grinding each HPFRCC mixture at the age of 90 days.

## 3. Results and Discussion

### 3.1. Shrinkage of HPFRCCs

#### 3.1.1. Effect of EXA Type

**Figure 1:**
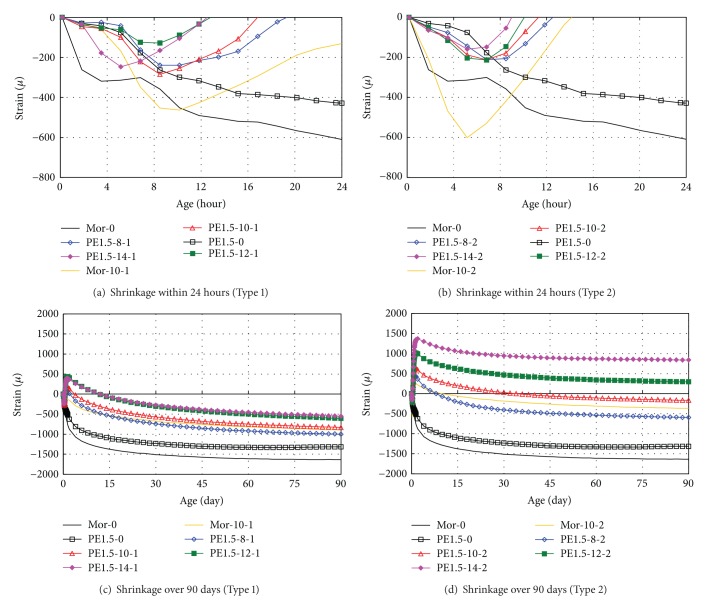
Shrinkage.

Figures 1(a) and 1(b) show the shrinkage for each specimen over a 90-day period. As the results for the amount of shrinkage change, the expansion rate after shrinkage in the early stages increases in increments of the replacement rate of the EXA. Eventually, the total amount of shrinkage is reduced.

Figures 1(a) and 1(b) show the amount of shrinkage within 24 hours after placing the HPFRCC. The use of EXAs remarkably reduces early age shrinkage. The experimental results confirm that the early age shrinkage of HPFRCC mixtures is less than that of mortar (*Mor* in figures). Shrinking occurs in the Type 1 specimens mostly during the 5 hours of placement, and then expansion begins after approximately 8 hours. Specimen PE15-14-1 initiates expansion 3 hours earlier than the other specimens, which is due to the substantial increase in the hydration reaction rate of the specimen with 14% EXA.

Figure 1(b) shows that the Type 2 specimens subsequently expand as a result of an increase in the replacement rate after 4 hours of placement. Similarly, the Type 1 specimens expand with an increase in the replacement rate due to the fast reaction rate of the ettringite.

After early age shrinkage, the expansion rate of the Type 2 specimens is twice as high as that of the Type 1 specimens. This result is due to the amount of ettringite that has formed [19].

Once the expansion strain of 700 *μ* is exceeded, as seen in Figure 1(d), the amount of shrinkage decreases significantly compared to the rate of expansion. Even after 90 days, specimens PE1.5-12-2 and PE1.5-14-2 are still under expansion pressure.

#### 3.1.2. Effect of Fiber Reinforcement

The Mor-0 and PE1.5-0 specimens, with and without reinforcing fiber, respectively, show a reduction of 200 *μ* for early age shrinkage, as seen in Figures 1(a) and 1(b). These findings confirm that this shrinkage reduction is due to the reinforced fiber in the HPFRCC mixtures [19].

#### 3.1.3. Effect of EXA Replacement Levels

**Figure 2:**
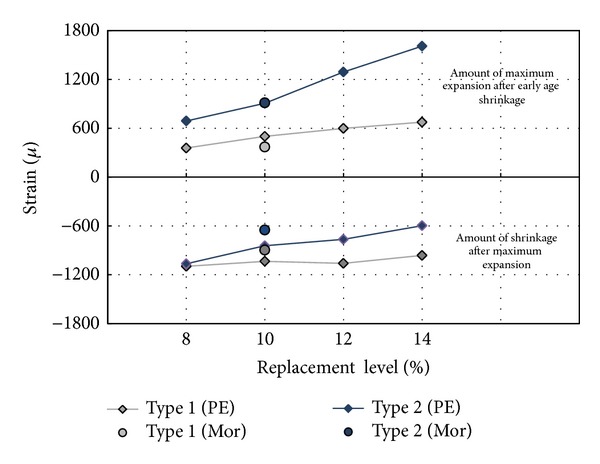
Shrinkage depending on type and replacement rate of expansion admixture.

Figure 2 shows the final shrinkage results for the specimens with the replacement rates (8% to 14%) of the admixtures, as measured after 90 days. For the Type 1 specimens, the expansion strains are in the range of 357~674 *μ*. For the Type 2 specimens, the expansion strains are in the range of 689~1608 *μ*, which are twice as high as the expansion factors of the Type 1 specimens. 

The final shrinkage results after maximum expansion are in the range of −1096~−962 *μ* and −1064~−597 *μ* for the Type 1 and Type 2 specimens, respectively. In short, the Type 2 specimens have higher final expansion strain levels after early shrinkage and less shrinkage at the end (after maximum expansion) than the Type 1 specimens. This result is due to the fact that an unexpected amount of ettringite forms under the same replacement rate for both Type 1 and Type 2 specimens. Thus, diverse mechanical properties are expected in the HPFRCC matrix.

### 3.2. Compressive Performance

**Figure 3:**
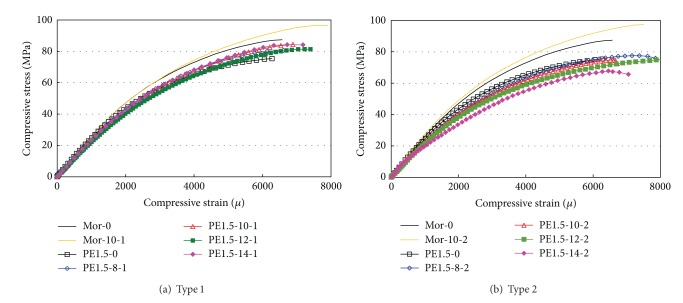
Compressive stress versus strain relationships: (a) Type 1 and (b) Type 2.

Figure 3 shows the stress versus strain relationship that determines the compressive strength and elastic modulus values. The figure shows only up to the maximum stress level. The results are summarized in Table 4.

The Type 1 HPFRCC specimens show similar stiffness values up to 80% of the compressive strength. The Type 2 HPFRCC specimens tend to exhibit reduced stiffness with an increase in the replacement rate, as seen in Figure 3. The internal hardened cement matrix loosens due to expansion.

**Figure 4:**
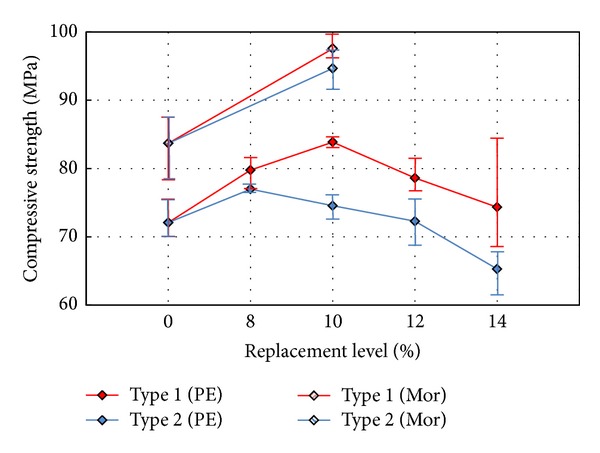
Comparison of compressive strength values.

Figure 4 shows the average compressive strength values for each replacement rate of the EXAs. The highest compressive strength values are obtained for specimen PE1.5-10-1, which has an expansion factor of 500 *μ*, and for specimen PE1.5-8-2, which has an expansion factor of 700 *μ*. The highest compressive strength values for the PE1.5-10-1 and PE1.5-8-2 specimens are 84 MPa and 77 MPa, respectively. However, the compressive strength tends to decrease when using the replacement rate that exceeds that of the specimen with the highest compressive strength value. This result is due to excessive expansion in the HPFRCC mixture. Therefore, the allowable stress limits of the fiber in the HPFRCC mixture are exceeded.

**Figure 5:**
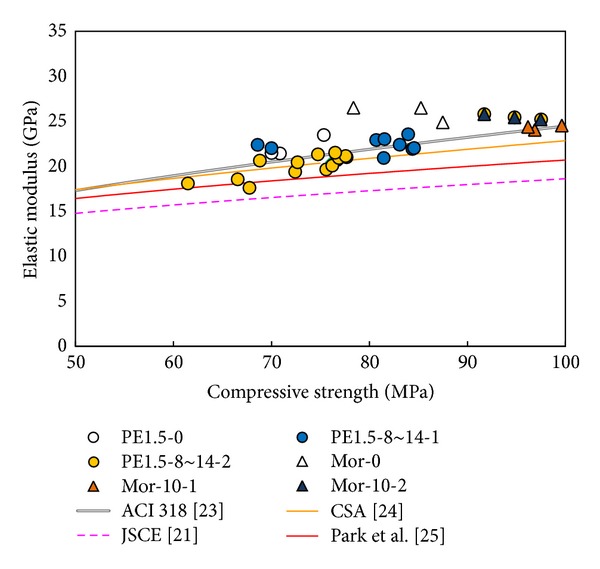
Compressive strength versus elastic modulus values.

The chord modulus in this study is used to define the elastic modulus in accordance with ASTM C469 [22].  Figure 5 shows a comparison between the compressive strength and the chord modulus values. In addition, the graph includes the predicted curves for the elastic modulus that are proposed by the American Concrete Institute (ACI) 318-08 [23], Canadian Standards Association (CSA) A23.304 [24], Japan Society of Civil Engineer (JSCE) [21], and Park et al. [25]. The predicted equations for the elastic modulus are listed in Table 5.

The HPFRCC elastic modulus values obtained from the ACI and CSA equations, which are computed using the unit weight (*w*_*c*_) of the HPFRCC mixtures and compressive strength (*f*_*c*_′), match well with the experimental results, although the equations provided by the JSCE and Park that include the effects of the HPFRCC properties slightly underestimate the experimental results. Overall, however, these equations can be used to determine the elastic modulus values of the HPFRCC mixtures.

### 3.3. Flexural Performance

Table 6 shows the results of the flexural tests. The initial flexural stiffness values are similar for all the specimens. With fiber reinforcement, the flexural strength of the specimen, regardless of the replacement amount of the EXA, is three times higher than that of the control specimen. The flexural strength increases by approximately 20% with a change in the replacement rate of the EXA in the HPFRCC mixtures. In the case of specimen PE1.5-10-1, a 57% increase in flexural strength, 22 MPa, is obtained. In the case of specimen PE1.5-8-2, a 24% increase in flexural strength, 17.4 MPa, is obtained.

In a comparison of the shrinkage and compressive strength test results, replacement rates of approximately 10% for the Type 1 EXA and approximately 8% for the Type 2 EXA show promising results.

**Figure 6:**
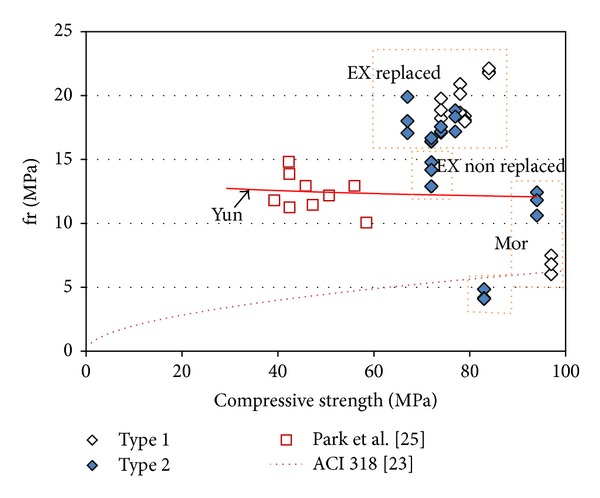
Compressive strength versus modulus of rupture values.

Figure 6 shows the compressive strength versus modulus of rupture values. The figure also includes the predicted values given by the authors [19] and those found in the ACI specifications (*fr* = 0.63√(*f*_*c*_′)(*MPa*)) [23]. The experimental results for the specimens without an EXA match well with the predicted results. When adding an EXA, a higher modulus of rupture value is obtained under the same compressive strength as without an EXA. This finding shows that flexural strength possibly can be improved by adding an EXA.

**Figure 7:**
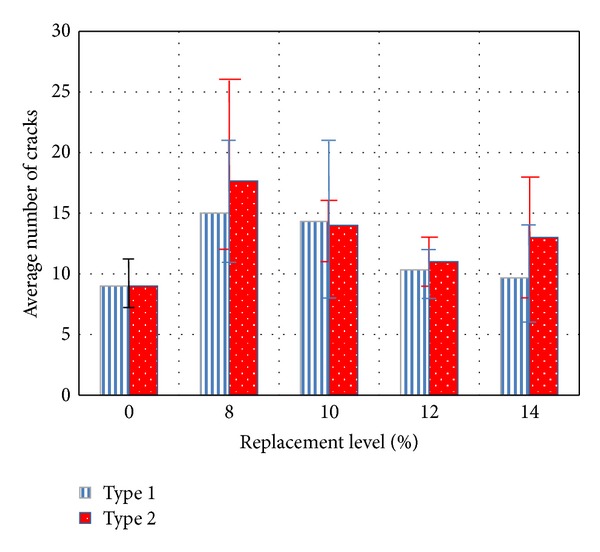
Average number of cracks obtained from flexural tests.

Figure 7 presents the averaged maximum and minimum numbers of cracks with respect to the replacement rate of the EXA. All the specimens containing fiber failed; the figure shows the stress distribution in terms of micro-cracking. In the case of the replacement EXA, the number of cracks decreases, and the distribution capacity improves compared to the HPFRCC mixtures without an EXA.

The highest number of cracks at the maximum load is obtained for specimen PE1.5-10-1 (replacement level of 10%) and specimen PE1.5-8-2 (replacement level of 8%), which indicates that effective stress redistribution is expected for both specimens.

### 3.4. Direct Tensile Performance

**Figure 8:**
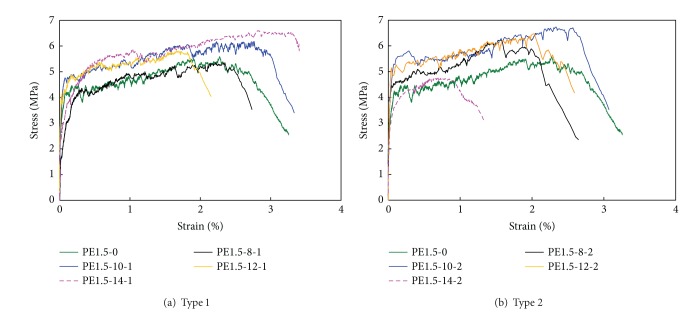
Tensile stress versus strain relationships.

Figure 8 shows the average stress and strain relationships of the tensile specimens for each representative specimen. The Type 2 specimens tend to have less tensile stress and strain-hardening effects than the specimens without an EXA. Among them, specimen PE1.5-8-2 shows the highest initial stiffness and maximum tensile stress values. The strain-hardening effects start at the strength value, which is 10% higher than that of the other specimens.

Using the same replacement rates for compressive strength and flexural strength (10% for Type 1 and 8% for Type 2), relatively good tensile strength is obtained. The strain-hardening effects observed in the Type 2 specimens that exhibit high early age shrinkage are relatively fewer than those found for the Type 1 specimens. This finding is due to the fact that the development of tensile strength is prohibited by the high level of expansion in the HPFRCC matrix that contains the Type 2 EXA.

In short, the strain-hardening effects might not be considerable in the Type 2 specimens, but they are significant in specimen PE1.5-10-1. The high expansion rate of the Type 2 specimens could be somewhat helpful in developing initial stiffness [26]. 

The direct tensile test results are summarized in Table 7. The maximum strain is determined at the time of 80% maximum strength after reaching maximum strength.

### 3.5. Cracking Behavior

**Figure 9:**
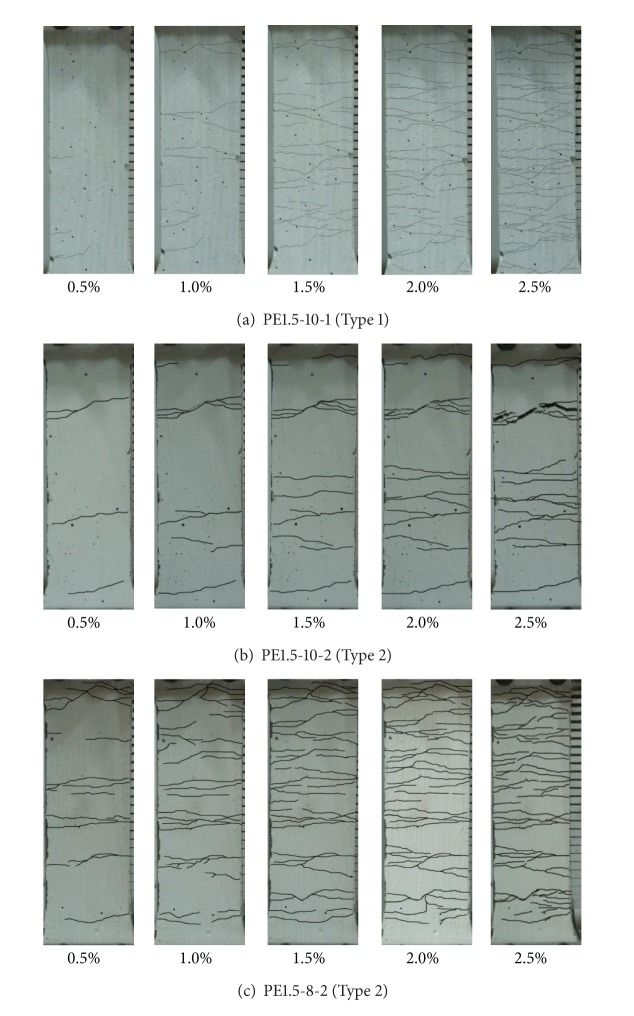
Crack propagation during direct tensile tests.

**Figure 10:**
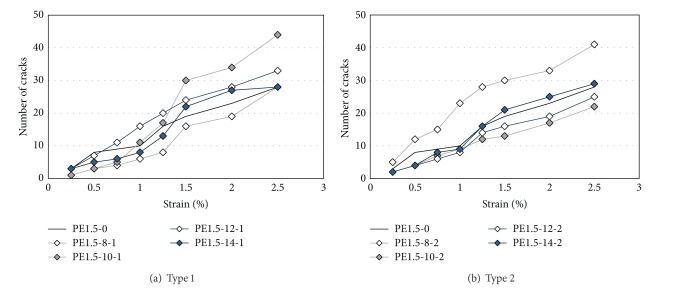
Number of cracks during tensile tests.

The numbers of cracks and crack patterns with respect to the level of deformation are shown quantitatively in Figures 9 and 10.

The crack width in most of the specimens tends to increase once 1% tensile strain is reached. For the Type 1 specimen with 10% replacement rate (PE1.5-10-1), the cracks in the HPFRCC mixture are dissipated widely, as shown in Figure 9(a). However, the cracks in the Type 2 specimens are scattered locally, as shown in Figure 9(b), except for specimen PE1.5-8-2. For specimen PE1.5-8-2, the distribution of the cracks is similar to that of the Type 1 specimens, as shown in Figure 9(c). In short, the different replacement rates of the EXAs are expected, depending on the type of EXA that is used to control cracking.

Figure 10 shows the number of cracks that is likely in each specimen for each type of EXA. A relatively large number of cracks is observed in the Type 1 specimens. Generally, for specimens with high tensile strength values, a large number of cracks is observed for both cases.

### 3.6. Scanning Electron Microscope (SEM)

**Figure 11:**
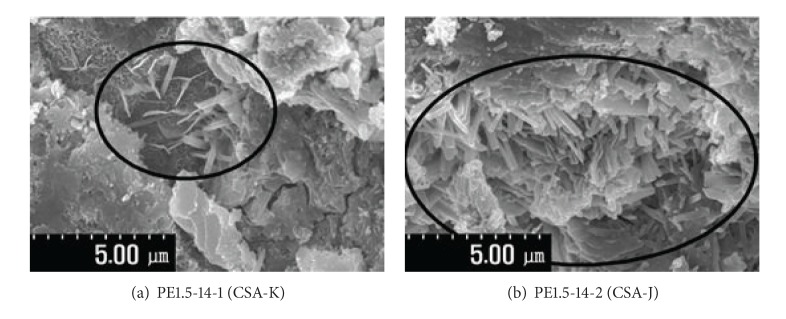
Formation of ettringite (SEM analysis).

Figure 11 shows that more ettringite forms with an increase in the replacement rate of EXA regardless of type. However, with the same replacement rate, ettringite forms locally inside the voids in the case of the Type 1 specimens. In the case of CSA-J (Type 2) specimens, the ettringite forms more densely in the voids than in the Type 1 specimens. This relative density with the same replacement rate of EXA may be due to the inclusion of F-CaO in the CSA EXA.

## 4. Performance Index of HPFRCC Mixtures

**Figure 12:**
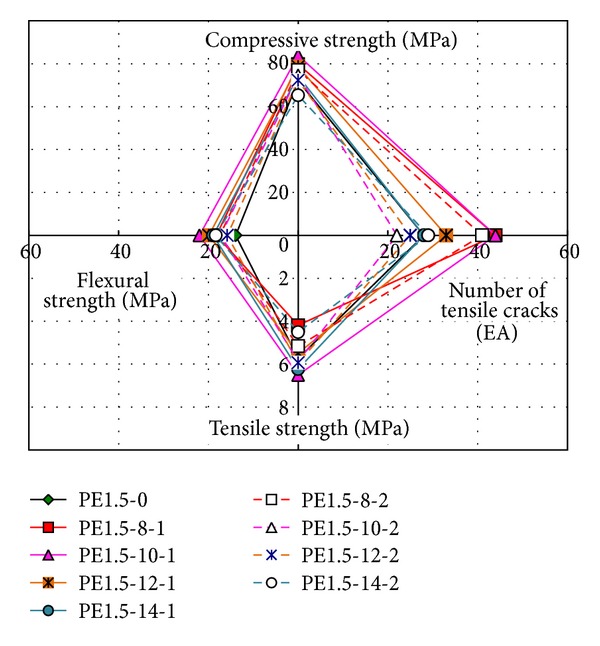
Performance index for HPFRCC mixtures.

**Figure 13:**
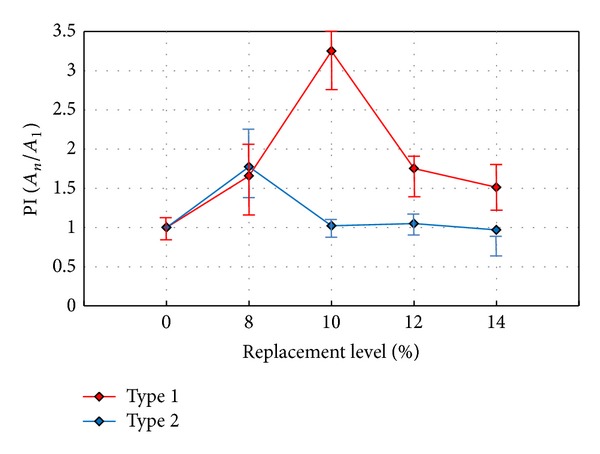
Comparison of performance index results with respect to replacement level.

To determine the mechanical performance of HPFRCC mixtures that contain EXAs, a performance index (PI) is adopted in this study. The PI represents an area composed of four points obtained from compressive strength, flexural strength, and tensile strength tests and the number of tensile cracks at failure. The comparisons for each specimen are presented in Figures 12 and 13. An important parameter that is used to determine the overall performance of the HPFRCC mixtures is crack dissipation, which is found from direct tensile testing. That is, the number of tensile cracks at failure is included to represent the crack dissipation in the HPFRCC mixtures. 

Figure 13 shows a comparison of the averaged PI values for the HPFRCC mixtures without an EXA. The proper replacement rate with respect to type of EXA can be determined quantitatively using the PI values.

## 5. Conclusions

Based on this limited experimental study, several conclusions are drawn regarding the effects of CSA on the mechanical properties of certain HPFRCC mixtures, including the various types and replacement rates of EXAs, as indicated below.

1. After early age shrinkage, the Type 2 specimens expand twice as much as the Type 1 specimens, because the F-CaO in the Type 2 EXA forms ettringite.
2. Compressive strength tends to increase incrementally when an EXA is used as replacement. For the Type 1 specimens, the specimens with 10% replacement show the highest compressive strength with an expansion of 500 *μ* (5% higher than the control specimen). For the Type 2 specimens, the specimens with 8% replacement show the highest compressive strength with an expansion of 700 *μ* (10% higher than the control specimen). When the replacement rates of 10% (for Type 1) and 8% (for Type 2) are exceeded, the compressive strength tends to decrease.
3. Based on a comparison of the failure modes used in the flexural tests, the performance of crack dispersion and stress redistribution in the HPFRCCs that contain an EXA is improved. For both the Type 1 and Type 2 specimens, a reliable relationship of flexural stress versus displacement is observed with the replacement rates of 10% and 8%, respectively. A flexural strength of 30% to 40% is higher than that in the control specimen.
4. The tensile strength and strain values are similar, regardless of the type of EXA used in the HPFRCC mixtures. The tensile strength of specimen PE1.5-10-1 increases 10% more than that of the control specimen. Specimen PE1.5-8-2 in the Type 2 group of EXAs has the highest tensile strength, which is similar to that of the control specimen. For specimen PE1.5-14-2, the excessive expansion represents a negative effect on the compression strength but an insignificant effect on the tensile strength.
5. Improved mechanical properties are obtained with specimen PE1.5-10-1 and specimen PE1.5-8-2. The expansion values of around 500~700 *μ* for both the Type 1 and Type 2 specimens play a role in positively affecting the mechanical properties.
6. This study is limited by the types of EXAs examined. Thus, additional parameters (e.g., water-to-cement ratio, design compressive strength, and batch mix design) need to be considered to determine the appropriate amount of EXA replacement.

## Figures and Tables

**Table 1:** Chemical components of CSA-based expansion admixtures (EXAs).

EXA (type)	Specific gravity (kg/m^3^)	Specific surface (cm²/g)	Chemical composition						
			SiO_2_	Al_2_O_3_	Fe_2_O_3_	CaO	MgO	SO_3_	F-CaO
CSA-K (Type I)	2.9	2,280	1.48	13.10	0.60	47.80	0.50	32.27	—

CSA-J (Type 2)	2.9	3,117	3.80	13.55	0.30	51.35	—	28.66	16.02

**Table 2:** Physical properties of PE fiber.

Fiber	Specific gravity (kg/m^3^)	Length (mm)	Diameter (*μ*m)	Aspect ratio	Tensile strength (MPa)	Elastic modulus (GPa)
PE	0.97	12	12	1,250	2,500	75

**Table 3:** Mix proportions of HPFRCCs.

Mixture type	W/B (%)	Replacement ratio^1^ (%)	Fiber vol. (%)	Unit weight (kg/m^3^)						
				cement	water	sand	EXA^2^	PE^3^	MC^4^	SP^5^
Mor-0	30	0	0	1,300	390	520	0	0	0.52	16.0
Mor-10		10		1,166	389	518	130	0	0.52	16.7
PE1.5-0		0	1.5	1,281	384	512	0	14	0.52	16.0
PE1.5-8*		8		1,175	383	511	102	14	0.52	16.7
PE1.5-10		10		1,149	383	511	128	14	0.52	16.7
PE1.5-12		12		1,123	383	510	153	14	0.52	16.0
PE1.5-14		14		1,096	382	510	178	14	0.52	15.0

*PE1.5-8: PE (fiber type), 1.5 (fiber volume fraction), 8 (replacement level of EXA); ^1^replacement rate:

^2^expansive admixtures (CSA-K and CSA-J); ^3^polyethylene fiber; ^4^methyl cellulose; ^5^superplasticizer.

**Table 4:** Summary of compressive strength test results.

EXA (type)	Mixture type	Maximum strength (MPa)				Strain at maximum strength (%)				Elastic modulus (GPa)			
		No. 1	No. 2	No. 3	Ave.	No. 1	No. 2	No. 3	Ave.	No. 1	No. 2	No. 3	Ave.
None	Mor-0	85	87	78	84	0.55	0.66	0.46	0.56	26.5	24.9	26.5	26.0
	PE1.5-0	75	71	70	72	0.66	0.55	0.61	0.61	23.5	21.4	21.5	22.1

CSA-K (Type 1)	Mor-10	97	100	96	98	0.77	0.71	0.67	0.72	24.1	24.5	24.4	24.3
	PE1.5-8	81	77	82	80	0.65	—	0.63	0.64	22.9	—	23.0	23.0
	PE1.5-10	85	84	83	84	0.70	0.65	0.69	0.68	22.0	23.5	22.4	22.7
	PE1.5-12	77	81	78	79	0.70	0.73	0.62	0.68	20.7	20.9	21.0	20.9
	PE1.5-14	69	70	84	74	0.54	0.42	0.68	0.55	22.4	22.0	21.9	22.1

CSA-J (Type 2)	Mor-10	95	97	92	95	0.68	0.75	0.67	0.68	25.4	25.2	25.8	25.5
	PE1.5-8	77	78	76	77	0.76	0.72	0.71	0.73	20.9	21.1	21.5	21.2
	PE1.5-10	73	75	76	75	0.71	0.65	0.76	0.71	20.5	21.3	20.1	20.6
	PE1.5-12	69	76	72	72	0.79	0.88	0.75	0.80	20.6	19.6	19.5	19.9
	PE1.5-14	67	68	61	65	0.71	0.65	0.53	0.63	18.6	17.6	18.1	18.1

**Table 5:** Predicted equations for the elastic modulus.

ACI 318 [23]	$E_{c}={w_{c}}^{1.5}0.043\sqrt{f_{c}^{\prime }}$
CSA [24]	$E_{c}=\left(3300\sqrt{f_{c}^{\prime }}+6900\right){\left(\frac{w_{c}}{2300}\right)}^{1.5}$
JSCE [21]	$E_{c}=1.77\times 10^{4}\times \sqrt{\left(\frac{w_{c}}{18.5}\right)}\times {\left(\frac{f_{c}^{\prime }}{60}\right)}^{1/3}$
Park et al. [25]	$E_{c}={w_{c}}^{1.5}0.057\sqrt[{3}]{f_{c}^{\prime }}$

**Table 6:** Summary of flexural test results.

EXA (type)	Mixture Type	Modulus of rupture (MPa)				Deflection at peak load (mm)			
		No. 1	No. 2	No. 3	Ave.	No. 1	No. 2	No. 3	Ave.
None	Mor-0	4.1	4.2	4.8	4.4	0.04	0.04	0.05	0.04
	PE1.5-0	14.2	12.9	14.8	14.0	1.18	1.10	0.64	0.97

CSA-K (Type 1)	Mor-10	6.8	7.5	6.0	6.8	0.07	0.08	0.07	0.07
	PE1.5-8	18.0	—	18.4	18.2	1.80	—	2.36	2.08
	PE1.5-10	22.1	21.8	—	22.0	1.98	1.95	—	1.97
	PE1.5-12	20.1	18.7	20.9	19.9	1.31	1.93	2.07	1.77
	PE1.5-14	18.9	19.8	18.2	19.0	1.33	1.19	1.88	1.47

CSA-J (Type 2)	Mor-10	6.1	5.2	5.8	5.7	0.07	0.05	0.06	0.06
	PE1.5-8	18.8	15.2	18.3	17.4	1.80	1.04	1.73	1.52
	PE1.5-10	17.1	17.2	17.6	17.3	1.14	1.23	1.30	1.22
	PE1.5-12	16.4	15.4	15.7	15.8	1.24	0.78	1.14	1.05
	PE1.5-14	19.9	17.1	18	18.3	1.65	1.00	1.84	1.50

**Table 7:** Summary of direct tensile test results.

EXA (type)	Mixture type	Maximum strength (MPa)						Tensile strain at peak load (%), (strain at 80% maximum strength after maximum strength (%))					
		1	2	3	4	5	Ave.	1	2	3	4	5	Ave.
None	PE1.5-0	6.16	6.67	5.49	5.58	4.61	5.70	3.30 (3.82)	3.80 (4.32)	2.16 (2.83)	2.27 (2.80)	1.03 (1.55)	2.51 (3.06)

CSA-K (Type 1)	PE1.5-8	2.90	3.19	6.94	4.03	5.35	4.48	0.22 (0.41)	0.10 (0.33)	— (−)	0.40 (0.77)	— (−)	0.24 (0.50)
	PE1.5-10	4.47	6.17	7.29	5.36	6.05	5.87	2.53 (2.77)	2.23 (3.08)	2.30 (2.65)	1.53 (1.99)	2.18 (3.19)	2.16 (2.74)
	PE1.5-12	4.41	4.85	4.58	5.81	5.81	5.09	0.53 (0.82)	1.09 (1.67)	0.57 (1.27)	0.97 (1.35)	1.66 (2.04)	0.96 (1.43)
	PE1.5-14	6.30	5.24	5.48	5.80	6.59	5.88	1.20 (1.28)	1.86 (2.22)	1.44 (1.64)	1.67 (1.83)	— (−)	1.54 (1.74)

CSA-J (Type 2)	PE1.5-8	4.27	6.22	5.20	4.08	—	4.94	0.39 (0.54)	1.53 (2.15)	0.02 (1.06)	0.29 (0.91)	—	0.56 (1.165)
	PE1.5-10	6.74	4.59	3.88	5.44	5.44	5.22	2.33 (2.76)	0.36 (0.86)	0.82 (1.10)	0.58 (1.33)	0.88 (1.43)	1.00 (1.45)
	PE1.5-12	5.23	6.48	5.72	4.86	5.62	5.58	0.4 (1.07)	2.03 (2.38)	0.78 (1.32)	1.33 (2.16)	1.15 (1.77)	1.14 (1.74)
	PE1.5-14	4.27	4.5	4.75	4.20	3.26	4.20	0.47 (0.90)	0.91 (1.16)	0.69 (1.18)	0.32 (0.63)	— (−)	0.60 (0.97)
